# The genetic diabetic nurse: transforming diabetes care

**DOI:** 10.1186/1472-6955-14-S1-K1

**Published:** 2015-10-08

**Authors:** Maggie Shepherd

**Affiliations:** 1 Honorary Clinical Professor, University of Exeter Medical School, Exeter, EX1 2LU, UK

## 

Monogenic diabetes (diabetes caused by a change in a single gene) accounts for around 2% of all diabetes but is not well recognised and is initially misdiagnosed in 80-90% of cases. This frequently leads to inappropriate treatment and advice including unnecessary use of insulin injections. This presentation illustrates how nursing research has increased our understanding of the impact of a genetic diagnosis of diabetes and treatment change from insulin injections to sulphonylurea tablets for individuals and their families.

Rapid advances in genetic technology have resulted in the identification of 30 genes causing monogenic diabetes. Identifying a means of effectively translating these genetic findings into diabetes clinical care was clearly needed. Training experienced Diabetes Specialist Nurses as Genetic Diabetes Nurses across the UK has increased awareness and recognition of monogenic diabetes, leading to improvements in clinical care. The role of the Genetic Diabetes Nurse, in the education of other health care professionals and aiding identification of patients with monogenic diabetes, and how this could be used as a model for other areas is discussed.

